# Infection-Related Adverse Events of Tisagenlecleucel in Pediatric Relapsed/Refractory B-Cell Acute Lymphoblastic Leukemia: A FAERS Pharmacovigilance Study

**DOI:** 10.3390/children13081054

**Published:** 2026-08-07

**Authors:** Xiaoxiao Song, Yaohua Liu, Xiaohong Qiao

**Affiliations:** Department of Pediatrics, Tongji Hospital, Tongji University School of Medicine, 389 Xincun Road, Shanghai 200065, China; 2211629@tongji.edu.cn (X.S.); 2532334@tongji.edu.cn (Y.L.)

**Keywords:** tisagenlecleucel, relapsed/refractory B-cell acute lymphoblastic leukemia, pediatrics, infections and infestations, FDA Adverse Event Reporting System (FAERS)

## Abstract

**Highlights:**

**What are the main findings?**

- This first Food and Drug Administration Adverse Event Reporting System pharmacovigilance study revealed a 30.28% infection-related adverse event reporting proportion among pediatric relapsed/refractory B-cell precursor acute lymphoblastic leukemia patients receiving tisagenlecleucel, with infected patients exhibiting a 40.27% mortality rate; most infections arose within one month while a small fraction occurred over one year later.
- Clostridioides difficile, influenza and adenovirus yielded significant disproportionate reporting signals suggestive of potential high-risk pathogens, and hypogammaglobulinemia/hypoxia showed higher co-occurrence rates with infections than classic chimeric antigen receptor T-cell(CAR-T) toxicities (cytokine release syndrome, immune effector cell-associated neurotoxicity syndrome).

**What are the implications of the main findings?**

- Clinicians should implement rigorous short-term surveillance in the first 15 days alongside long-term extended follow-up to address both early and delayed infectious complications.
- Targeted prophylaxis against identified high-risk pathogens warrants consideration; our data provides a hypothesis-generating basis for future standardized infection risk stratification in pediatric CAR-T treatment.

**Abstract:**

**Background**: Tisagenlecleucel (tis-cel) is the sole chimeric antigen receptor T-cell (CAR-T) therapy approved for pediatric/adolescent relapsed/refractory B-cell acute lymphoblastic leukemia (r/r B-ALL). Infection-related adverse events (IRAEs) represent a leading cause of non-relapse mortality, yet dedicated analysis in patients <18 years remains limited. **Objective**: This study aimed to analyze the characteristics of IRAEs in pediatric and adolescent patients with r/r B-ALL treated with tis-cel, based on the FDA Adverse Event Reporting System (FAERS) database. The analysis included the reporting proportion, temporal distribution, pathogen spectrum, and overlapping features with other adverse events, generate hypotheses for infection prevention and control in patients aged <18 years. **Research Design and Methods**: We analyzed FAERS data (August 2017–March 2025) and included 492 cases of <18-year-old r/r B-ALL patients treated with tis-cel. Disproportionality analyses (reporting odds ratio, ROR; information component, IC), time-to-onset analysis, and co-occurrence assessments were performed on 149 infection-related reports. **Results**: Infection-related AEs occurred in 30.28% of cases, with significantly higher mortality in infected versus non-infected patients (40.27% vs. 12.83%, *p* < 0.001). Most infections (93.86%) occurred within one month (median time-to-onset = 4 days), peaking within 15 days (77.50%); 2.65% occurred after one year. Significant disproportionate reporting signals were observed for Clostridioides difficile (ROR_025_ = 5.45), influenza virus (ROR_025_ = 7.16), and adenovirus (ROR_025_ = 3.51). Hypogammaglobulinemia (52.94%) and hypoxia (54.90%) exhibited higher co-occurrence with infections than CAR-T-specific toxicities (23.08–35.41%). **Conclusions**: Infection-related AEs following tis-cel treatment are frequent and associated with significantly increased mortality in pediatric and adolescent r/r B-ALL patients. While most occur early, late infections warrant long-term vigilance. Disproportionality analyses identified signals suggestive of potential high-risk pathogens such as Clostridioides difficile,, influenza, and adenovirus, and hypogammaglobulinemia/hypoxia may serve as early warning indicators. These hypothesis-generating findings require validation in prospective studies.

## 1. Introduction

Currently, improvements in risk-stratified antileukemic therapy and supportive care have increased the overall survival (OS) of children with B-cell precursor acute lymphoblastic leukemia (BCP-ALL) to nearly 95%. However, outcomes following conventional chemotherapy and hematopoietic stem cell transplantation have reached a plateau, with approximately 15–20% of patients eventually experiencing relapse or developing refractory disease [1,2]. Chimeric antigen receptor T-cell (CAR-T) therapy has emerged as a major novel treatment option for children, adolescents, and young adults with r/r B-ALL [3].

Tisagenlecleucel (tis-cel) is the first approved CAR-T product, receiving FDA approval in 2017 for the treatment of patients up to 25 years of age with BCP-ALL in second or later relapse [1]. It remains, to date, the only CAR-T therapy approved in the pediatric setting for r/r B-ALL.

In the landmark ELIANA trial, tis-cel demonstrated an overall response rate of 81%, with 12-month event-free survival (EFS) of 50% and overall OS of 76% in pediatric patients with B-ALL [4]. These results led to the approval of tis-cel for the treatment of patients up to 25 years of age with r/r B-ALL Subsequent data from both clinical trials and real-world studies have shown complete response rates ranging from 73% to 92%, 12-month EFS between 31% and 69%, and OS between 63% and 86% [2,5,6,7,8,9,10], confirming tis-cel as a highly promising treatment for r/r B-ALL.

However, safety concerns associated with this therapy require urgent attention, particularly infectious complications, which have emerged as the leading cause of non-relapse mortality—accounting for 50% of non-relapse mortality events. In contrast, CAR T-cell-specific toxicities such as cytokine release syndrome (CRS), immune effector cell-associated neurotoxicity syndrome (ICANS)/neurotoxicity, and hemophagocytic lymphohistiocytosis (HLH) represent only minor drivers of non-relapse mortality [11,12,13].

Current research on infections following tis-cel treatment often combines data from adult populations. Although systematic studies focusing specifically on individuals <18 years old remain limited, emerging evidence underscores the urgency of this issue. A recent study [2] reported that among 125 pediatric and adolescent patients with r/r B-ALL treated with tis-cel, the infection rate reached 28.5%. The median age of the enrolled population was 11 years (range: 6.5–15.9 years). Another study [10] focusing on children under 3 years of age with r/r B-ALL found that among 35 patients receiving tis-cel, the infection rate was 29.41%. However, these studies have certain limitations. First, they only describe basic characteristics of infections and do not thoroughly investigate key aspects such as the temporal distribution of infections, risk differences among various pathogens, or the overlapping associations between infections and other toxicities, thereby failing to fully support the optimization of clinical prevention and control strategies. Second, both studies have relatively small sample sizes and narrow age ranges, making it difficult to comprehensively reflect the overall situation of the <18-year-old patient population. Furthermore, as small-scale studies, they lack multi-center, large-sample data support, which limits the generalizability of their findings. Therefore, in-depth analysis of post-CAR-T infections in the <18-year-old population is of significant clinical value and research necessity.

The FDA Adverse Event Reporting System (FAERS), as one of the largest spontaneous reporting databases globally, provides crucial resources for identifying rare adverse events [14]. To address these key gaps, this study utilizes real-world data from FAERS to conduct, for the first time, a pharmacovigilance analysis of IRAEs associated with tis-cel treatment in <18-year-old patients with r/r B-ALL. The research aims to elucidate the pathogen spectrum, temporal distribution, outcomes, and overlapping features with other toxicities of IRAEs following tis-cel treatment, to identify potential safety signals, and thereby to provide a hypothesis-generating evidence base for optimizing infection prevention and control strategies in pediatric patients.

## 2. Materials and Methods

### 2.1. Data Sources and Extraction

This study is a retrospective disproportionality analysis utilizing the FAERS. FAERS is a publicly accessible post-marketing drug safety surveillance database that contains a large number of adverse event (AE) reports. Each entry in the database provides detailed information, including patient demographic characteristics, suspected drugs, AE descriptions, patient outcomes, time to onset of the adverse event, and reporting country, among other details. It serves as a vital resource for post-marketing drug safety monitoring and has been updated quarterly since 2004 [15,16]. An AE report is classified as serious if it results in death, is life-threatening, requires hospitalization or prolongs existing hospitalization, causes permanent disability, or leads to congenital anomalies or birth defects [11].

FAERS data were retrieved from quarterly data extraction files, which are publicly accessible at https://fis.fda.gov/extensions/FPD-QDE-FAERS/FPD-QDE-FAERS.html (accessed on 24 April 2025). The FAERS database consists of seven distinct datasets, including: demographic and administrative information (DEMO), drug information (DRUG), patient outcomes (OUTC), reporting sources (RPSR), therapy start and end dates for reported drugs (THER), drug indications (INDI), and adverse event terms coded in MedDRA (REAC).

We extracted all case records from the FAERS database between August, 2017 (the date when tis-cel was approved by the FDA for the treatment of patients <18 years of age with r/r B-ALL) and March 2025. To ensure data quality and avoid redundancy, data preprocessing and cleaning were performed prior to analysis. Duplicate records were removed and data were refined following official FDA recommendations, retaining only the most recent CASE_ID based on the latest FDA_DT. Only cases where the drug role code was designated as “Primary Suspect (PS)” were included [16,17].

The inclusion criteria for this study were predefined as follows: (1) patients aged <18 years at the time of adverse event reporting, identified through the AGE field in the DEMO table, with age units standardized to years; (2) tis-cel designated as the primary suspect (PS) drug in the DRUG table; (3) a documented indication consistent with B-cell acute lymphoblastic leukemia, ascertained from the INDI table; and (4) reports sub-mitted between 30 August 2017 and 31 March 2025.

To operationalize criterion (3), MedDRA Preferred Terms (PTs) in the INDI dataset were categorized into three hierarchical groups to balance sensitivity with specificity: (i) definitive B-ALL phenotypes: B precursor type acute leukemia and B-cell type acute leukemia; (ii) explicit relapsed or refractory disease states: acute lymphocytic leukemia refractory, acute lymphocytic leukemia recurrent, and leukemia recurrent; and (iii) non-specific or supportive terms: acute leukemia, acute lymphocytic leukemia, lymphocytic leukemia, and lymphocyte adoptive therapy. However, owing to the inherent limitations of spontaneous reporting systems—including incomplete case narratives and the absence of structured treatment-line data—relapsed/refractory status could not be definitively con-firmed at the individual case level. Instead, relapsed/refractory status was inferred from the overall clinical context: tis-cel is exclusively approved for r/r B-ALL in patients ≤ 25 years, and its designation as the primary suspect drug in patients < 18 years provides reasonable indirect evidence that the treated condition fell within the approved indication. Cases in tier (iii) were retained when this contextual inference was compatible with pediatric r/r B-ALL.

Exclusion criteria included duplicate records, cases with missing age or indication data, reports where tis-cel was listed as a concomitant or interacting drug rather than the PS, and, specifically for time-to-onset analyses, cases with input errors.

Adverse events in FAERS are classified and coded using the Medical Dictionary for Regulatory Activities (MedDRA), version 27.1. We identified relevant cases by querying the “Infections and Infestations” System Organ Class (SOC) and extracted all associated High-Level Group Terms (HLGT), High-Level Terms (HLT), and Preferred Terms (PT). During data mining, the www.drugbank.ca (accessed on 24 April 2025) database was used as a reference to standardize and verify the generic and brand names of tis-cel.

**Figure 1:**
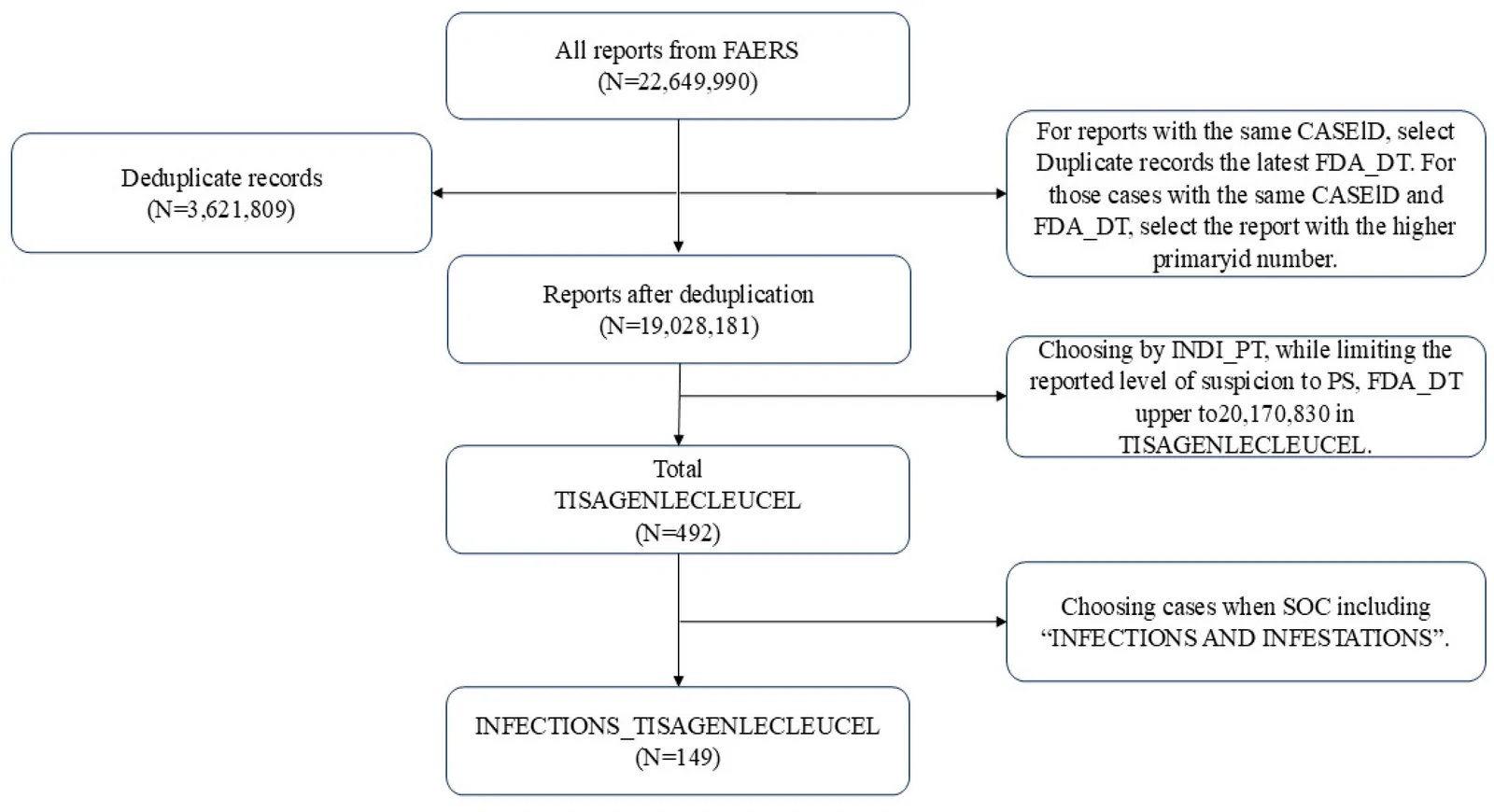
Flowchart of Case Selection for Tisagenlecleucel-Associated Infection and Infestation Adverse Events. FAERS, FDA Adverse Event Reporting System; PS, primary suspect; SOC, System Organ Class; PT, Preferred Term.

From an initial total of 2,264,999 AE reports retrieved from FAERS, a final set of 149 infection-related cases was identified. The detailed data screening process is illustrated in Figure 1.

As FAERS is a publicly available and anonymized database, neither institutional review board approval nor patient informed consent was required for this study.

### 2.2. Data Analysis

#### 2.2.1. Primary Outcome Analysis

This study employed disproportionality analysis methods to explore potential associations between adverse events and tis-cel, an approach widely used in pharmacovigilance research. To enhance the robustness of adverse drug reaction correlation assessments, we selected ROR, IC, and their respective lower limits of the 95% confidence intervals (95% CI) as evaluation metrics. Additionally, reports with fewer than three cases were excluded to minimize bias from false-positive results.

The ROR method, known for its high sensitivity, is suitable for preliminary screening of potential signals. The IC method, based on Bayesian principles, offers greater robustness and specificity. The combined use of both methods improves the sensitivity of signal detection while effectively reducing the false positive rate, thereby enhancing the reliability and stability of safety signal identification [18]. The formulas for these algorithms, corresponding thresholds, and signal detection criteria for each method are summarized in Table 1 [18]. A significant signal was determined if all the following conditions were met: first, the lower limit of the 95% CI for ROR (ROR_025_) exceeded 1; second, the lower limit of the 95% CI for IC (IC_025_) exceeded 0; and third, at least three reports were available. Generally, higher values of these metrics indicate stronger signals, reflecting a more pronounced correlation between the drug and the adverse event. All data processing and statistical analyses were performed using R software version 4.3.1 and Microsoft Excel 2019.

#### 2.2.2. Secondary Outcome Analysis

To characterize the temporal pattern of IRAEs following tis-cel treatment, we analyzed the proportion of patients experiencing such events over different post-treatment periods. Cases with input errors (e.g., EVENT_DT earlier than START_DT) or incomplete records were excluded to ensure data accuracy.

The co-occurrence rate (also referred to as concomitant reporting) was defined as the simultaneous reporting of an AE classified under the “Infections and Infestations” System Organ Class and an AE from any other SOC within the same case report. This metric is based on the co-reporting of both types of events within a single case and does not require temporal overlap or a specific time window between the events [14]. The primary objective of the co-occurrence analysis is to systematically identify and quantify combinations of IRAEs and non-infection SOC events reported together in the same cases, thereby highlighting high-risk clinical symptom complexes that warrant particular attention.

#### 2.2.3. Statistical Methods

Categorical variables (e.g., gender, age groups) were compared using the chi-square test, and continuous variables (e.g., age) were summarized using median with interquartile range. Relative risk (RR) with its 95% confidence interval was calculated to quantify risk differences between groups. All statistical tests were two-sided, and a *p*-value < 0.05 was considered statistically significant. Disproportionality analyses were conducted using self-programmed functions in R (version 4.3.1). The reporting odds ratio (ROR) was estimated using the standard case-non-case design, while the information component (IC) was computed using the classical IC formula under a Bayesian framework. The 95% CIs for ROR were calculated using the asymptotic delta method, and the 95% credibility intervals for IC were derived from the posterior distribution. Disproportionality statistics for events with zero cell counts were treated as missing to avoid bias from continuity correction.

## 3. Results

### 3.1. Characteristics of Infection-Related Adverse Event Reports Following Tisagenlecleucel in the FAERS Database

This study analyzed data from the FAERS database between 30 August 2017, and 31 March 2025. After screening, a total of 492 adverse event reports involving patients < 18 years old with r/r B-ALL, in which tis-cel was designated as the primary suspect (PS) drug, were included (hereinafter referred to as the “overall treatment group”). Among these, 149 cases (30.28%, 149/492) contained terms related to the Medical Dictionary for Regulatory Activities (MedDRA) SOC “Infections and Infestations” (hereinafter referred to as the “infection-related group”). The remaining cases in the overall treatment group, excluding the infection-related group, were categorized as the “non-infection-related group.”

The median age of patients in the infection-related group was 10 years (Q1–Q3: 5–14 years), similar to that of the overall treatment group (10 years, Q1–Q3: 6–14 years). Detailed demographic and clinical characteristics of the patients are presented in Table 2.

Demographic characteristics showed that in the infection-related group, male patients accounted for 61.07% (91/149) and females for 37.58% (56/149), which was consistent with the sex distribution in the overall treatment group (males: 55.28%; females: 40.65%). The rate of infection-related events was higher in male patients (33.46%, 91/272) than in female patients (28.00%, 56/200), with male patients having a 20% higher risk of IRAEs (RR = 1.20, 95% CI: 0.89–1.61). However, this difference was not statistically significant (*p* = 0.21).

Regarding age distribution, the infection rates were as follows: <3 years old: 33.33% (13/39); 3–5 years old: 37.88% (25/66); 6–11 years old: 24.86% (45/181); and ≥12 years old: 32.04% (66/206). No statistically significant differences were observed among age groups (χ^2^ = 5.26, *p* = 0.154).

In terms of reporting sources, reports from the United States accounted for 44.31% (218/492) and those from Japan for 21.34% (105/492) in the overall treatment group. The majority of infection-related adverse event reports originated from the United States (83.89%, 125/149) and Japan (4.70%, 7/149).

Analysis of clinical outcomes revealed that the infection-related group exhibited a higher reported mortality rate (RR = 3.14, 95% CI: 2.20–4.49). The mortality rate in the infection-related group (40.27%, 60/149) was 3.14 times higher than that in the non-infection-related group (12.83%, 44/343) (χ^2^ = 45.20, *p* < 0.001), accounting for 57.69% (60/104) of all reported deaths. Life-threatening events occurred in 9.40% (14/149) of the infection-related group, representing 28.00% (14/50) of all life-threatening events in the overall cohort, highlighting the importance of vigilant infection monitoring and management following CAR-T therapy, while acknowledging that FAERS data do not permit definitive causal attribution. No statistically significant differences were observed between the two groups in other outcome measures, such as disability, life-threatening conditions, or permanent complications requiring intervention (*p* > 0.05).

### 3.2. Disproportionality Analysis of Tisagenlecleucel-Associated Infections and Infestations Based on Preferred Terms

This study evaluated the signal strength of infections and infestations associated with tis-cel treatment in patients <18 years old with relapsed/refractory B-ALL using disproportionality analysis. Preferred Terms with at least 3 reported cases were included (37 PTs in total), representing an overall reporting volume of 266 cases. The top 10 most frequently reported adverse event PTs related to tis-cel treatment in the target population were: Clostridioides difficile infection (n = 17, 6.39%), Staphylococcal infection (n = 16, 6.02%), Sinusitis (n = 15, 5.64%), Adenovirus infection (n = 13, 4.89%), Rhinovirus infection (n = 12, 4.51%), Influenza (n = 12, 4.51%), Candida infection (n = 10, 3.76%), Gastrointestinal infection (n = 10, 3.76%), Enterococcal infection (n = 9, 3.38%), Cellulitis (n = 9, 3.38%).

**Figure 2:**
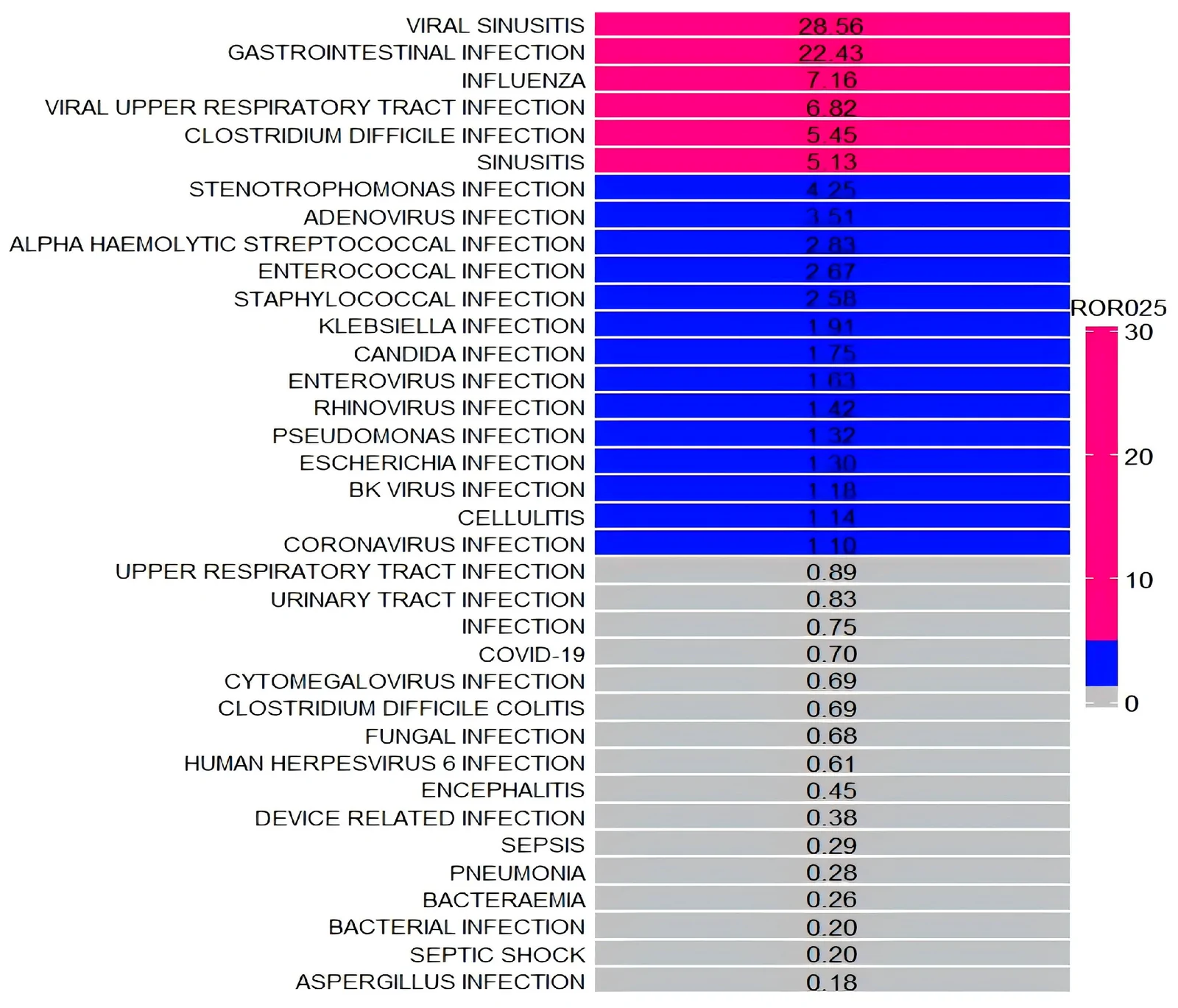
Safety Signal Heatmap Based on ROR_025_ ROR, reporting odds ratio; ROR_025_, lower limit of the 95% confidence interval for ROR. Grey, blue and red color blocks correspond to ascending ROR_025_ values in turn. Signals were considered significant if ROR_025_ > 1, IC_025_ > 0, and N ≥ 3 (see Table 1 for detailed criteria).

To visually represent the differences in signal strength of infection events, Figure 2 presents a safety signal heatmap based on ROR_025_ values. The results show that viral sinusitis (ROR_025_ = 28.56), gastrointestinal infection (ROR_025_ = 22.43), influenza (ROR_025_ = 7.16), and viral upper respiratory tract infection (ROR_025_ = 6.82) exhibited significantly higher signal strengths compared to other events. Additionally, Clostridioides difficile infection (ROR_025_ = 5.45) and adenovirus infection (ROR_025_ = 3.51) also demonstrated clear signals.

A total of 15 significant signals were identified using the combined threshold criteria (ROR_025_ > 1 and IC_025_ > 0) (see Table 3 for details). Among these, five events demonstrated strong associations: viral sinusitis (ROR_025_ = 28.56, IC_025_ = 1.41), Clostridioides difficile infection (ROR_025_ = 5.45, IC_025_ = 1.63), gastrointestinal infection (ROR_025_ = 22.43, IC_025_ = 1.71), influenza (ROR_025_ = 7.16, IC_025_ = 1.58), and sinusitis (ROR_025_ = 5.13, IC_025_ = 1.52).

In addition, although adenovirus infection (ROR_025_ = 3.51, IC_025_ = 1.16) and staphylococcal infection (ROR_025_ = 2.58, IC_025_ = 0.95) did not meet the strict combined threshold, they still warrant enhanced monitoring and vigilance due to their disproportionality signals and reporting frequency. However, it should be emphasized that several signals meeting the combined threshold—including viral sinusitis (n = 8), Stenotrophomonas infection (n = 4), and alpha hemolytic streptococcal infection (n = 4)—were derived from very few reports. While these events reached statistical significance, their limited reporting volume increases the risk of spurious associations and overestimated effect sizes, necessitating cautious interpretation. These findings provide important signal clues for clinical surveillance and prevention of infections associated with CAR-T cell therapy, although their clinical significance warrants further investigation.

### 3.3. Signals of Tisagenlecleucel Treatment-Associated Infections and Infestations Based on HLT and HLGT

Further classification and evaluation of potential infectious pathogens induced by tis-cel treatment revealed that, at the HLGT level, the top three types of pathogen infections are shown in Table 4. Bacterial infectious diseases were the most frequently reported (n = 80, 5.68%), followed by viral infectious diseases (n = 60, 4.26%) and fungal infectious diseases (n = 22, 1.56%). Based on the analysis of 266 reports at the Preferred Term (PT) level and 247 reports at the HLT level (see Table 5 for details), bacterial infectious diseases showed a significant risk signal at the HLGT level (ROR_025_ = 1.67, IC_025_ = 0.24). Among these, Clostridium infections at the HLT level were most prominent (n = 26, 10.53%; ROR_025_ = 2.01, IC_025_ = 0.65). In contrast, viral infections overall did not reach significance at the HLGT level (ROR_025_ = 1.02, IC_025_ = −0.14). This may be due to the dilution effect caused by the inclusion of lower-risk viral types (e.g., herpesvirus, IC_025_ = −1.16) masking the signals of stronger pathogens such as influenza virus.

Further cross-level validation revealed consistent high-risk signals for Clostridioides difficile infection at both the PT level (17 cases, 6.39%; ROR_025_ = 5.45, IC_025_ = 1.63) and the HLT level (IC_025_ = 0.65). Similarly, influenza virus infection showed mutually supportive signals at the PT level (12 cases, 4.51%; ROR_025_ = 7.16, IC_025_ = 1.58) and the HLT level (ROR_025_ = 4.99, IC_025_ = 1.29). Adenovirus infection also demonstrated associations across both levels (HLT level: ROR_025_ = 2.25, IC_025_ = 0.72; PT level: ROR_025_ = 3.51). Notably, although staphylococcal infection showed a risk signal at the PT level (n = 16, 6.02%; IC_025_ = 0.953), its signal attenuated at the HLT level (IC_025_ = 0.28). A similar discrepancy was observed for enterococcal infection, with a stronger signal at the PT level (IC_025_ = 0.76) than at the HLT level (IC_025_ = 0.40), suggesting that terminology classification may influence signal interpretation. It is noteworthy that gastrointestinal infection, as an independent event, exhibited a significant signal at the PT level (10 cases, 3.76%; ROR_025_ = 22.43, IC_025_ = 1.71). Due to the lack of a directly corresponding HLT term, it should be independently considered a high-risk event. In summary, Clostridioides difficile, influenza virus, and adenovirus were confirmed as significant high-risk pathogens through dual-level validation, warranting the highest priority in infection prevention and control. Enhanced monitoring is recommended for staphylococcal and enterococcal infections, while unexplained gastrointestinal infections require targeted preventive strategies.

The reporting rate of Fungal Infectious Disorders following tis-cel treatment was 1.56% (22/1409). Signal detection metrics indicated that this association did not reach the threshold for statistical significance (ROR_025_ = 0.32, IC_025_ = −1.38), suggesting that current data do not support a significant association between tis-cel and the risk of fungal infections.

### 3.4. Time-to-Onset Analysis of Infection Events

**Figure 3:**
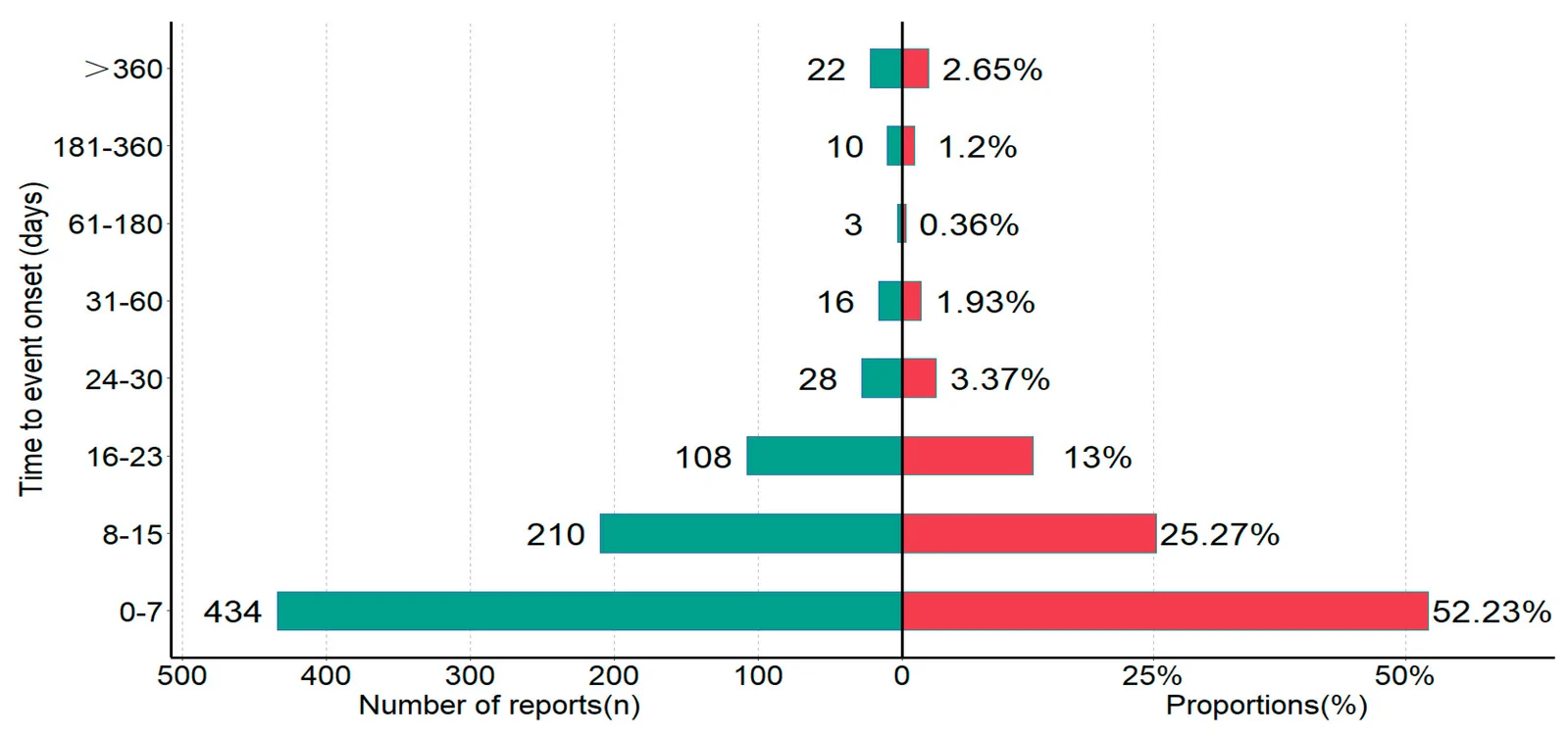
Temporal Distribution Characteristics of IRAEs Following Tisagenlecleucel Treatment The y-axis represents the proportion of IRAEs occurring within each time interval; the denominator for each percentage is the total number of IRAE reports (831) with documented time-to-onset.

Figure 3 presents the time-to-onset (TTO) analysis of PTs classified under the SOC “Infections and Infestations” following tis-cel treatment. This study included 831 PT reports with accurate and complete time records. The median TTO was 4 days (interquartile range: 1–9 days), indicating a distinct temporal pattern.

On the day of treatment, 125 IRAEs were reported (15.04% of all cases). Within the first month post-treatment, a total of 780 infection-related events (93.86%) were documented, significantly higher than in other periods and demonstrating clear temporal clustering. A more detailed breakdown shows that the first peak occurred within 0–7 days, with 434 cases (52.23%), followed by a second peak during 8–15 days, with 210 cases (25.27%). Cumulatively, 644 cases (77.50%) occurred within the first 15 days post-treatment, far exceeding the 136 cases (16.37%) reported between days 16 and 30.

Notably, although the reporting proportion of events gradually decreased starting from the second month, 2.65% of IRAEs (corresponding to the >360-day period) occurred more than one year after treatment, highlighting the need for long-term infection monitoring and follow-up in patients receiving tis-cel therapy.

### 3.5. Analysis of Co-Occurrence Rates Between IRAEs and Other Adverse Events Following Tisagenlecleucel Treatment

**Figure 4:**
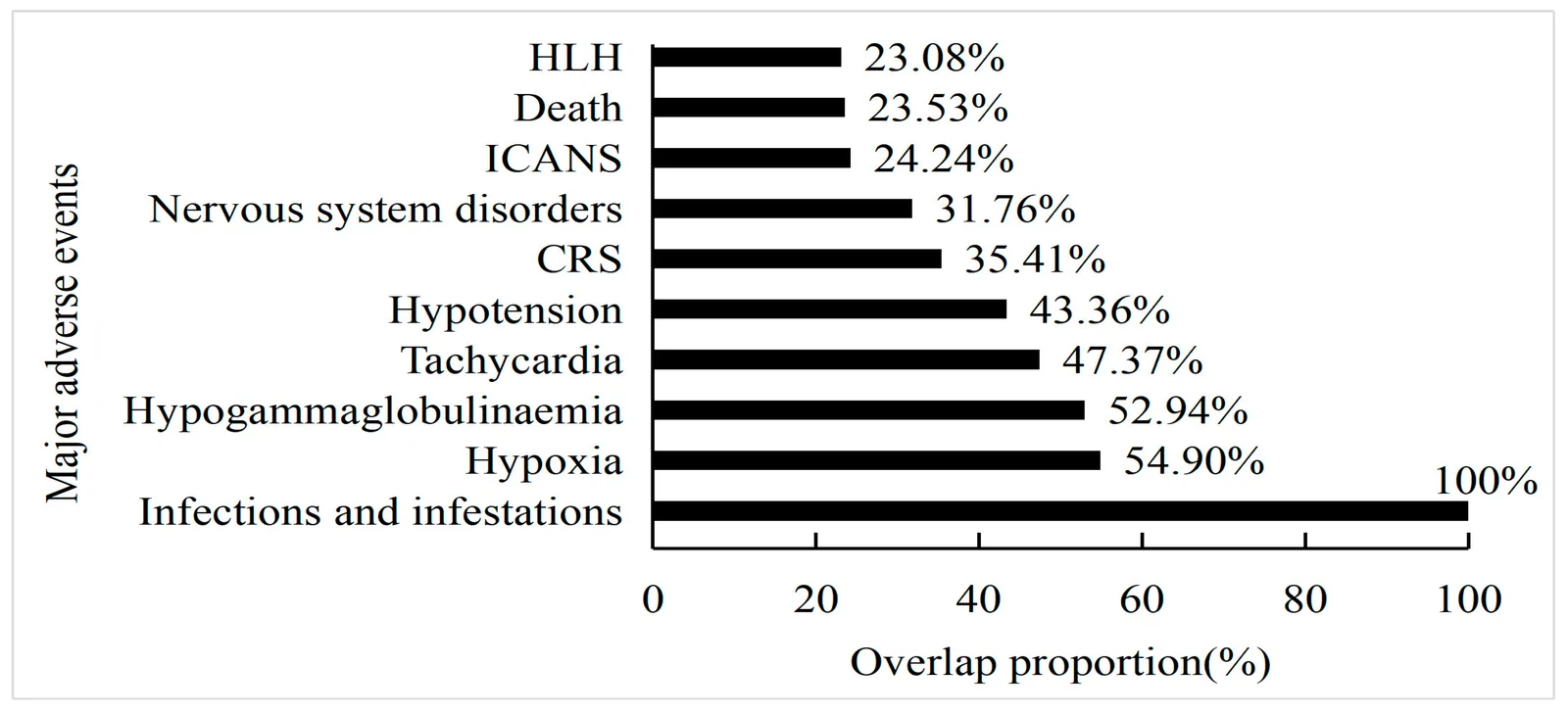
Infection-Related Adverse Events and Their Co-Occurrence with Other Toxicities Following Tisagenlecleucel Treatment. CRS, cytokine release syndrome; ICANS, immune effector cell-associated neurotoxicity syndrome; HLH, hemophagocytic lymphohistiocytosis. Co-occurrence rate is defined as the proportion of IRAE reports that also contained a specific non-infection AE term within the same case report. The denominator for all percentages is the total number of IRAE reports (149).

The analysis of co-occurrence proportions between IRAEs and other adverse events is shown in Figure 4. “Infections and infestations” served as the reference event in this study (co-occurrence rate: 100%), while the co-occurrence rates of other adverse events with infections varied. Specifically, the overlap rates between IRAEs and CAR-T-specific toxicities-CRS, ICANS, and HLH-were 35.41%, 24.24%, and 23.08%, respectively. The co-occurrence rates of infections with nervous system disorders, tachycardia, hypotension, hypogammaglobulinemia, and hypoxia were 31.76%, 47.37%, 43.36%, 52.94%, and 54.90%, respectively. Additionally, the overlap rate between infections and death was 23.53%.

## 4. Discussion

Tisagenlecleucel is the only CAR-T cell therapy approved for the treatment of pediatric and adolescent patients with r/r B-ALL. While it significantly improves treatment outcomes, infectious complications following therapy have become a major challenge limiting its clinical safety. Utilizing real-world data from the FAERS database, this study is the first to systematically characterize IRAEs in children and adolescents with r/r B-ALL treated with tis-cel. The results revealed an infection-related adverse event reporting rate of 30.28%, with a significantly higher mortality rate in the infection group (40.27%) compared to the non-infection group (12.83%, *p* < 0.001), underscoring the substantial impact of infections on patient prognosis. The temporal analysis showed a bimodal distribution: 93.86% of infections occurred within the first month after infusion (median TTO = 4 days), with 77.50% concentrated within the first 15 days. However, 2.65% of infections were delayed (>1 year), highlighting the need for a phased monitoring system. Through multidimensional signal detection, this study identified Clostridioides difficile (ROR_025_ = 5.45), influenza virus (ROR_025_ = 7.16), and adenovirus (ROR_025_ = 3.51) as high-risk pathogens with significant associations, providing a precise basis for targeted preventive strategies. Furthermore, the co-occurrence rates of hypogammaglobulinemia (52.94%) and hypoxia (54.90%) with infections were significantly higher than those of CAR-T-specific toxicities (CRS/ICANS/HLH: 23.08–35.41%), suggesting that these two indicators may serve as early warning markers for infection risk. These findings not only deepen the understanding of infectious complications following tis-cel therapy but also provide important evidence-based support for optimizing infection prevention and control strategies in pediatric and adolescent patients with r/r B-ALL.

### 4.1. High Reporting Proportion and Mortality of Infection-Related Adverse Events

This study found that among 492 pediatric patients (<18 years old) with r/r B-ALL treated with tis-cel, the overall reporting proportion of IRAEs was 30.28% (149/492). Among these, death occurred in 40.27% of cases with IRAEs (60/149), accounting for 57.69% (60/104) of all deaths in the cohort. Compared to the non-infection group, the mortality rate was significantly higher in the infection-related group (40.27% vs. 12.83%, *p* < 0.001). Additionally, 9.40% of infection-related events (14/149) were life-threatening, representing 28.00% (14/50) of all life-threatening events in the entire cohort. Although the FAERS database does not allow definitive attribution of the direct cause of death (which may involve infection, disease progression, or multifactorial overlap), the observed co-reporting of infections with higher reported mortality highlights the importance of vigilant infection monitoring and management following CAR-T therapy.

### 4.2. Comparison with Previous Studies

An international multicenter retrospective cohort study [10] focusing on children with r/r B-ALL who were <3 years old at screening reported an infection rate of 29% (10/34) after tis-cel treatment, which is consistent with the findings of the present study. In that study, 24% (8/34) of cases experienced grade ≥ 3 severe infections. Most current studies on tis-cel treatment for r/r ALL include children and young adults ≤ 25 years of age, with reported infection rates showing some variability. A recent multicenter study [2] involving 125 patients with r/r B-ALL (median age 11 years, range 0.7–25.7 years) reported an infection rate of 28.46%, with 60% being grade ≥ 3 severe infections, aligning closely with our results. A phase I study [19] of 25 pediatric patients with r/r B-ALL (age range 1–22.5 years, median 13.5 years) found that 36% of cases developed infections (24% were grade ≥ 3), and 12% were accompanied by severe neutropenia. A retrospective analysis [9] of 181 patients with r/r B-ALL (median age 12 years, range 0–26 years) from 15 U.S. institutions showed that 40.33% experienced at least one post-infusion infectious complication. The phase II ELIANA trial [20], which included 75 patients with r/r CD19-positive ALL (age range 3–23 years, median 11 years), reported an infection rate of 42.67% within 8 weeks after infusion (24% grade ≥ 3), with two infection-related deaths (2.7%); a subsequent safety update also documented one late fatal bacterial pulmonary infection. A pooled analysis [21] of 137 patients (median age 12 years, range 3–25 years) from the ELIANA and ENSIGN trials revealed an overall infection rate of 42.3% (19% grade ≥ 3), including viral infections in 13.9%, bacterial infections in 17.5%, and fungal infections in 5.8%. Among these, 35.8% of infections occurred within ≤4 weeks after infusion (14% grade ≥ 3), and 14.3% occurred between 4 and 8 weeks (8% grade ≥ 3). Another study [22] encompassing the ELIANA, ENSIGN, and B2101J phase II trials estimated a post-treatment infection rate of 65% in patients with r/r B-ALL.

Collectively, these studies indicate that the infection rate among children and young adults ≤ 25 years of age treated with tis-cel ranges from 36% to 65%, which is consistent with findings from large observational studies [23] reporting that 19–69% of patients receiving CD19-directed CAR-T cell therapy for hematologic malignancies develop infections. However, this range is higher than the 30.28% reporting proportion of IRAEs observed in the present study, which focused exclusively on patients < 18 years old. This discrepancy may be attributed to differences in the age ranges of the study populations.

Regarding fatal infections, a study by Ioannis Kyriakidis et al., [20] indicated that fatal infections accounted for 3.1–5% of the pediatric and young adult population treated with tis-cel. In contrast, among FAERS reports documenting IRAEs, 12.20% (60/492) also documented death, a proportion significantly higher than the previously reported range for infection-attributed mortality in prospective studies. This discrepancy may stem from: ① the inability of the FAERS database to distinguish the direct cause of death (infection, underlying disease, or other complications); and ② reporting bias, wherein severe infectious events are more likely to be reported. This study observed that the reported mortality rate was significantly higher in the infection-related group than in the non-infection-related group (40.27% vs. 12.83%, *p* < 0.001), while the rate of prolonged hospitalization was significantly lower in the infection-related group (16.78% vs. 24.49%, *p* = 0.049). This inverse association may be related to non-infectious factors such as early death in the infection-related group and the impact of underlying diseases shortening the hospital stay. Age distribution analysis showed no statistically significant difference in the reporting proportion of infection-related events across age groups (χ^2^ = 5.258, *p* = 0.154). However, the proportion of reported cases was relatively higher in patients aged 3–5 years (37.88%), which may be attributed to the immature immune system in this age group and further increased infection risk due to treatment-related thymic suppression. In terms of gender, the reporting proportion of infection-related events was slightly higher in males (33.46%) than in females (28.00%), but the difference was not statistically significant (*p* = 0.206). The current sample size may have been insufficient to detect potential age or gender associations, suggesting a need for future studies with larger samples or more refined stratification.

### 4.3. Temporal Distribution Characteristics and Mechanisms of Infection Occurrence

Infections post CAR-T therapy are typically classified as early (≤30 days) or late (>30 days) [24]. This study found 93.86% of IRAEs occurred within the first month, with 77.50% concentrated in the first 15 days-peaking in the first week (52.23%). Although infection rates declined after the first month, 2.65% of cases occurred beyond one year, emphasizing the critical need for intensified monitoring during the initial 30 days, especially the first two weeks, while maintaining long-term vigilance. This temporal distribution pattern is consistent with findings from multiple studies [23,24,25], which report a higher reporting proportion of infectious complications within the first month after treatment, followed by a gradual decline over subsequent months. Additionally, pediatric patients exhibit poorer tolerance to infections during the initial 90 days post-treatment, with 57% of infections being severe or life-threatening [26], highlighting the need for intensified early infection prevention and control measures in this population.

The high proportion of reported cases of early infections is closely linked to immunosuppression associated with CAR-T therapy. Patients often present with baseline immunodeficiency—including cytopenias, reduced marrow reserve, and hypogammaglobulinemia—due to underlying malignancies, prior anticancer treatments, or previous allogeneic HCT, which collectively elevate infection risk [20,23,24]. Prophylactic management of neutropenia and hypogammaglobulinemia before CAR-T infusion is essential to reduce early fatal infections. Lymphodepleting chemotherapy further intensifies immunosuppression, increasing susceptibility to opportunistic pathogens. A history of infection within 30–100 days before lymphodepletion is significantly associated with higher risk of early post-treatment infections [23,24]. In addition, treatment-related complications can exert additive effects on infection risk. Post-infusion immune dysregulation may trigger cytokine release syndrome (CRS), immune effector cell-associated neurotoxicity syndrome (ICANS), and HLH-like syndrome. The management of these complications, such as with tocilizumab or corticosteroids, further increases the risk of infections [23,27,28]. The CAR-T cell dose and the development of CRS also influence infection risk. Higher CAR-T cell doses (e.g., 2 × 10^7^ cells/kg) have been associated with increased infection risk [29], potentially due to a higher reporting proportion and severity of CRS. A study by Park et al. [30] confirmed that grade ≥3 CRS is an independent risk factor for infections (adjusted hazard ratio 2.67, *p* = 0.05), particularly elevating the risk of bloodstream infections.

Late-onset infectious events, particularly the 2.65% reporting proportion of infection-related events occurring beyond one year post-treatment, may be attributed to prolonged immunodeficiency induced by CD19-targeted CAR-T therapy. The “off-target effect” of CAR-T cells results not only in the elimination of malignant B cells but also in the destruction of normal B cells, leading to B-cell aplasia and persistent hypogammaglobulinemia, the duration of which remains uncertain [23,31].

### 4.4. Spectrum of High-Risk Pathogens

Based on disproportionality analyses, this study detected significant safety signals for Clostridioides difficile (ROR_025_ = 5.45), influenza virus (ROR_025_ = 7.16), and adenovirus (ROR_025_ = 3.51), suggesting these pathogens may serve as high-priority surveillance targets. Given the spontaneous reporting nature of FAERS data, the above findings reflect statistical associations and should be regarded as hypothesis-generating evidence rather than causal confirmation. Nevertheless, enhanced surveillance targeting these pathogens still holds clinical reference value. For staphylococcal and enterococcal infections, although they did not meet the combined threshold, enhanced monitoring is still recommended based on their disproportionality signals and reporting frequencies.

Most early infections were bacterial, with Clostridioides difficile colitis being the most frequently reported. Viral infections represented the second most common type, predominantly respiratory viruses [23,30,31]. Late infections were most often respiratory in origin [23]; some studies reported viral pathogens as the main cause [29,30], while others highlighted bacterial etiologies as remaining highly relevant [32,33].

Clostridioides difficile is a leading cause of infectious diarrhea worldwide, with approximately 20,000 pediatric cases reported annually—a number that continues to rise. Pediatric Clostridioides difficile infection can lead to severe complications such as pseudomembranous colitis, pneumatosis coli, toxic megacolon, bowel perforation, peritonitis, and multi-organ failure [34]. While metronidazole has traditionally been the first-line treatment, recent evidence supports oral vancomycin and fidaxomicin as safe and effective alternatives [35].

Early detection and aggressive antimicrobial intervention are crucial for reducing mortality associated with CAR-T-related infections. Influenza vaccination is recommended for all patients beyond 3 months post-CAR-T, regardless of immune reconstitution status. Although impaired immune recovery or ongoing immunosuppression may attenuate vaccine response, expert consensus maintains that vaccination can still reduce infection rates and improve outcomes [25].

Although viral sinusitis yielded a high ROR_025_ of 28.56 in this study, this signal was based on only 8 cases and may reflect a small-sample exaggeration effect. Similar caution applies to bacterial signals derived from sparse reports, such as Stenotrophomonas infection (n = 4) and alpha hemolytic streptococcal infection (n = 4), where small denominators increase the likelihood of chance findings and inflated ROR estimates. Consequently, these signals should be treated as exploratory rather than confirmatory, and further studies with larger samples are required to validate their true association with tis-cel therapy.

### 4.5. Hypogammaglobulinemia and Hypoxia as Frequently Co-Reported Features with Infections

This study utilized co-occurrence analysis to identify patterns of concurrent adverse events following tis-cel therapy, with the primary objective of identifying high-risk symptom clusters requiring heightened clinical attention. The analysis revealed that hypogammaglobulinemia had the highest overlap with IRAEs (52.94%), significantly exceeding that of cytokine release syndrome (CRS, 35.41%) and immune effector cell-associated neurotoxicity syndrome (ICANS, 24.24%). This finding is mechanistically consistent with the high infection reporting proportion, as B-cell depletion—caused by lymphodepleting chemotherapy and CAR-T cell therapy—is a well-established cause of hypogammaglobulinemia [23].

Notably, hypogammaglobulinemia (52.94%), hypotension (43.36%), and hypoxia (54.90%) demonstrated the strongest associations with infection-related events. While the cross-sectional nature of FAERS data precludes any determination of temporal sequence or predictive value, these high overlap rates suggest that concurrent presentation of these conditions with infections may be a clinically meaningful pattern. Accordingly, in patients presenting with these frequently co-reported features, clinicians should maintain a low threshold for infection screening and timely initiation of empirical anti-infective therapy, particularly when sepsis is suspected.

Furthermore, the temporal overlap and symptomatic similarities between CRS (onset within 1–14 days) and early infections [25] underscore the need for more specific diagnostic tools to avoid overtreatment. The 23.53% co-occurrence between infection-related events and death further emphasizes the critical importance of early recognition of concurrent complications, along with prompt initiation of prophylactic antibiotics, immunoglobulin replacement, and multidisciplinary interventions.

Age is a significant factor influencing the reporting proportion of hypogammaglobulinemia following CD19-targeted CAR-T cell therapy in pediatric patients. Due to delayed maturation of immunoglobulin subclasses and plasma cells, children exhibit a significantly higher proportion of reported cases, greater severity, and longer duration of hypogammaglobulinemia compared to adolescents or adults [4,36]. The reported reporting proportion varies widely across centers (20–90%), contributing to increased infection risk. However, the lack of standardized definitions and detection methods has prevented consensus on immunoglobulin G replacement therapy (IgG-RT) [37,38]. Some studies recommend initiating regular monthly IgG-RT at one month post-infusion or when IgG levels fall below 400 mg/dL [39]. If IgG remains below 400 mg/dL for three months with no improvement and is accompanied by severe or recurrent bacterial infections, particularly pulmonary infections, IgG-RT should also be initiated [40]. Doan et al. [41] suggest that IgG-RT be administered every 3–4 weeks when IgG is <400 mg/dL within 1–3 months post-infusion, continuing until age-appropriate normal IgG levels are achieved. For children under 10 years old, those with a history of pulmonary infection, or those receiving immunosuppressants, some studies recommend maintaining IgG > 800 mg/dL [42], while Arnold et al. [39] advocate for a threshold of >1000 mg/dL. Los-Arcos et al. [42] propose that replacement therapy should be continued in cases of B-cell aplasia or persistently low IgA/IgM. Discontinuation after B-cell recovery requires individualized assessment, as quantitative B-cell recovery does not always imply functional immune reconstitution, and IgG deficiency may persist. Therefore, immunologic evaluation should not rely solely on B-cell counts [26].

### 4.6. Fungal Infection Risk and Immune Management

The reporting proportion of fungal infectious adverse events in this study was 1.56%, with no significant risk signal detected. Notably, an IC_025_ value below zero (IC_025_ = −1.38) suggests that the reporting rate of fungal infections may be lower than the background expected level. However, this should be interpreted cautiously within the clinical context, as immunocompromised patients remain a high-risk population for fungal infections. However, the absence of a significant disproportionality signal does not constitute evidence of no causal association between tis-cel and fungal infections, particularly given that fungal pathogens often present with atypical or subtle clinical features, making them more challenging to diagnose compared to bacterial and viral infections [20]. Existing literature [43] indicates that the reporting proportion of yeast infections following CD19-targeted CAR-T therapy ranges from 1% to 10%, while mold infections occur in 0% to 7% of cases, predominantly within the first 30 days after infusion. The risk of fungal infection is closely associated with the degree of immunosuppression. Key risk factors include concurrent CRS, ICANS, HLH, use of tocilizumab/corticosteroids or other immunomodulators, prior treatment with more than five lines of therapy, and a history of allogeneic hematopoietic stem cell transplantation [20,23]. Prophylactic antifungal therapy is recommended for high-risk pediatric patients [37]. In clinical practice, enhanced fungal surveillance-such as serum galactomannan testing and PCR-based assays-should be implemented, especially for patients with prolonged immunosuppression or concurrent CRS.

### 4.7. Limitations

Despite utilizing the FAERS database to evaluate IRAEs associated with tis-cel in patients < 18 years old with r/r B-ALL and identifying valuable safety signals, this study has several limitations.

First, as a spontaneous reporting system, FAERS is susceptible to reporting bias, incomplete data, and duplicate entries. Although data cleaning and statistical adjustments were applied to mitigate these issues, residual confounding may still affect the accuracy of the results [16,17]. Importantly, the lack of a defined denominator in FAERS precludes estimation of true incidence; all reported proportions should be interpreted as reporting proportions rather than true occurrence rates.

Second, since FAERS is structured around drug-centric reporting, observed associations must be interpreted as signals for hypothesis generation rather than confirmatory evidence of causality. Critical clinical variables, including prior hematopoietic stem cell transplantation, lymphodepletion regimens, corticosteroid exposure, CRS severity, and baseline immune status, are not systematically captured. Consequently, these important residual confounders could not be adjusted for, and their influence on the observed associations cannot be ruled out. This limitation is inherent to spontaneous reporting systems but does not negate the value of identifying potential safety concerns that warrant prospective validation.

Third, because FAERS lacks structured cause-of-death adjudication, the observed co-occurrence of infections with fatal outcomes cannot be taken as definitive evidence that these deaths were attributable to infection. The direct cause of death in individual cases may reflect multifactorial contributions that cannot be reliably disentangled in spontaneous reporting systems. Similarly, the co-occurrence analysis is descriptive, reflecting adverse events documented concurrently within individual FAERS reports. Since FAERS lacks data on event timing, causal inferences are precluded. Nevertheless, the high overlap with hypoxia (54.90%) and hypogammaglobulinemia (52.94%) identifies clinically relevant risk clusters. These findings reinforce the need for clinicians to consider infection in the differential diagnosis when managing patients with these complications, pending validation of temporal relationships in prospective studies.

Fourth, the total number of FAERS reports for tis-cel in patients aged <18 years remains limited, and additional reports are needed to support comprehensive pharmacovigilance analyses. In particular, risk signals for viral sinusitis and certain bacterial infections were derived from very few cases, introducing considerable uncertainty regarding effect size estimation and generalizability—a limitation that should not be overlooked.

Finally, geographic and diagnostic heterogeneity may limit generalizability; further validation through international multicenter studies is recommended.

Critically, it should be particularly noted that the disproportionality analysis employed in this study is primarily intended for signal detection, and the identified associations can only serve as a basis for hypothesis generation and cannot be directly used to infer causality.

## 5. Conclusions

This study presents the first systematic analysis of IRAEs following tis-cel treatment in patients < 18 years old with r/r B-ALL using the FAERS database. Key findings include a high reporting proportion of infection-related events (30.28%), significantly elevated mortality in the infection group (40.27% vs. 12.83%, *p* < 0.001), distinct temporal dynamics, a defined pathogen spectrum, and co-occurrence patterns with other non-infectious toxicities. The results suggest that infections may represent an important factor associated with adverse post-CAR-T outcomes, though FAERS data preclude definitive causal attribution of mortality. Although most infections occurred early (within the first month), with a pronounced peak during the initial 15 days, a small but notable proportion (2.65%) manifested beyond one year post-treatment. This challenges the conventional focus on short-term monitoring and emphasizes the need for long-term follow-up to address delayed infectious risks. Disproportionate reporting signals for specific pathogens identified Clostridioides difficile, influenza virus, and adenovirus as high-risk surveillance targets, providing a hypothesis-generating basis for future targeted prophylactic strategies. Although these data are of considerable reference value, they should not be interpreted as causal evidence, and their clinical translational value warrants confirmation through prospective safety studies. Furthermore, high co-occurrence rates of infections with hypogammaglobulinemia and hypoxia highlight these conditions as frequently concomitant features that deserve clinical attention; however, the cross-sectional nature of the data precludes their use as predictive early warning indicators. These findings may inform hypothesis generation for future prospective studies rather than directly supporting risk stratification at this stage.

Despite the rich data source provided by FAERS, limitations such as potential reporting biases and the relatively small sample size of pediatric patients require cautious interpretation of the results. Future studies should expand the cohort, perform finer subgroup analyses, and incorporate multinational multicenter data to validate these findings. Approved in 2025, puzolcabtagene autoleucel, China’s first humanized CD19 CAR-T for pediatric r/r B-ALL, enables head-to-head comparative research to establish standardized infection monitoring and supportive care protocols for children.

## Figures and Tables

**Table 1:** Algorithms, Formulas, and Criteria for Assessing Adverse Event Signals in Pharmacovigilance Analysis.

Algorithms	Formulas	Criteria
ROR	$\mathrm{ROR}=\frac{\left(a/c\right)}{\left(b/d\right)}=\frac{ad}{bc}$	lower limit of 95% CI > 1, N ≥ 3
	$SE\left(lnROR\right)=\sqrt{\left(\frac{1}{a}+\frac{1}{b}+\frac{1}{c}+\frac{1}{d}\right)}$	
	$95\%\;CI{=}^{ln\left(\mathrm{ROR}\right)\pm 1.96\sqrt{\left(\frac{1}{a}+\frac{1}{b}+\frac{1}{c}+\frac{1}{d}\right)}}$	
IC	$IC=\log_{2}\left(\frac{a\left(a+b+c+d\right)}{\left(a+c\right)\left(a+b\right)}\right)$	lower limit of 95% CI > 0, N ≥ 3
	$95\%\;CI=E\left(IC\right)\pm 2\times \sqrt{V\left(IC\right)}$	

ROR, reporting odds ratio; IC, information component; ROR_025_, lower limit of the 95% confidence interval for ROR; IC_025_, lower limit of the 95% credibility interval for IC; N, number of reports for a given Preferred Term in tisagenlecleucel reports. a: reports with tis-cel and target AE; b: reports with tis-cel and other AEs; c: reports with other drugs and target AE; d: reports with other drugs and other AEs.

**Table 2:** Demographic and Clinical Characteristics of <18-Year-Old Patients with r/r B-ALL Treated with Tisagenlecleucel.

Characteristics	Infection-Related Group (N = 149) (N, %)	Overall Treatment Group (N = 492) (N, %)
GENDER		
Female	56 (37.58%)	200 (40.65%)
Male	91 (61.07%)	272 (55.28%)
Unknown	2 (1.34%)	20 (4.07%)
AGE_GROUP/(years)		
<3	13 (8.72%)	39 (7.93%)
3–5	25 (16.78%)	66 (13.41%)
6–11	45 (30.20%)	181 (36.79%)
≥12	66 (44.30%)	206 (41.87%)
Median_Age(Q1, Q3)	10(5, 14)	10(6, 14)
EVENT_YEAR		
2017	5 (3.36%)	18 (3.66%)
2018	18 (12.08%)	60 (12.20%)
2019	14 (9.40%)	53 (10.77%)
2020	7 (4.70%)	37 (7.52%)
2021	5 (3.36%)	35 (7.11%)
2022	11 (7.38%)	34 (6.91%)
2023	7 (4.70%)	31 (6.30%)
2024	2 (1.34%)	10 (2.03%)
2025	0 (0.00%)	1 (0.20%)
Unknown	80 (53.69%)	213 (43.29%)
REPORTER_COUNTRY		
United States	125 (83.89%)	218 (44.31%)
Japan	7 (4.70%)	105 (21.34%)
Other Countries	17(11.41%)	169 (34.35%)
REPORTERS		
Nurse	20 (13.42%)	64 (13.01%)
Healthcare Professional	15 (10.07%)	87 (17.68%)
Lawyer	0 (0.00%)	0 (0.00%)
Medical Doctor	103 (69.13%)	292 (59.35%)
Pharmacist	1 (0.67%)	6 (1.22%)
Other	10 (6.71%)	43 (8.74%)
Unknown	0 (0.00%)	0 (0.00%)
OUTCOMES		
Death	60 (40.27%)	104 (21.14%)
Disability	0 (0.00%)	3 (0.61%)
Hospitalization-Initial or Prolonged	25 (16.78%)	109 (22.15%)
Life-Threatening	14 (9.40%)	50 (10.16%)
Required Intervention to Prevent Permanent Impairment/Damage	0 (0.00%)	1 (0.20%)
Other Serious Event (Important Medical Event)	50 (33.56%)	200 (40.65%)
Unknown	0 (0.00%)	25 (5.08%)

**Table 3:** Signal Strength of Infections and Infestations Following Tisagenlecleucel Therapy Based on Preferred Terms.

PT	ROR	ROR_025_	IC	IC_025_	N (%)
VIRAL SINUSITIS	228.79	28.56	4.69	1.41	8 (3.01)
GASTROINTESTINAL INFECTION	71.78	22.43	4.38	1.71	10 (3.76)
INFLUENZA	14.39	7.16	3.28	1.58	12 (4.51)
VIRAL UPPER RESPIRATORY TRACT INFECTION	16.33	6.82	3.41	1.19	8 (3.01)
CLOSTRIDIOIDES DIFFICILE INFECTION	9.49	5.45	2.84	1.63	17 (6.39)
SINUSITIS	9.23	5.13	2.82	1.52	15 (5.64)
STENOTROPHOMONAS INFECTION	14.18	4.25	3.28	0.27	4 (1.50)
ADENOVIRUS INFECTION	6.45	3.51	2.42	1.16	13 (4.89)
ALPHA HAEMOLYTIC STREPTOCOCCAL INFECTION	8.72	2.83	2.78	0.16	4 (1.50)
ENTEROCOCCAL INFECTION	5.47	2.67	2.23	0.76	9 (3.38)
STAPHYLOCOCCAL INFECTION	4.40	2.58	1.95	0.95	16 (6.02)
KLEBSIELLA INFECTION	4.24	1.91	1.92	0.37	7 (2.63)
CANDIDA INFECTION	3.40	1.75	1.63	0.44	10 (3.76)
RHINOVIRUS INFECTION	2.58	1.42	1.27	0.27	12 (4.51)
PSEUDOMONAS INFECTION	2.88	1.32	1.42	0.05	7 (2.63)

N (%) represents the number and percentage of infection-related PT records out of the total PT records in the infection-related group (N = 266). ROR, reporting odds ratio; IC, information component; ROR_025_ and IC_025_ denote the lower limits of the 95% confidence intervals for ROR and IC, respectively. A statistically significant pharmacovigilance signal was defined as ROR_025_ > 1, IC_025_ > 0, and N ≥ 3.

**Table 4:** Analysis of Reporting Rates and Signal Strength of IRAEs Following Tisagenlecleucel Treatment Based on HLGT Classification.

HLGT_Name	N	%	ROR_025_	IC_025_
BACTERIAL INFECTIOUS DISORDERS	80	5.68	1.67	0.24
VIRAL INFECTIOUS DISORDERS	60	4.26	1.02	−0.14
FUNGAL INFECTIOUS DISORDERS	22	1.56	0.32	−1.38

HLGT, High-Level Group Term). Percentages (%) were calculated as the count of each HLGT category divided by the total number of eligible HLGT records under the SOC “Infections and Infestations” (1409). ROR_025_, lower limit of the 95% confidence interval for ROR; IC_025_, lower limit of the 95% credibility interval for IC. A statistically significant pharmacovigilance signal was defined as ROR_025_ > 1, IC_025_ > 0, and N ≥ 3.

**Table 5:** Analysis of Reporting Rates and Signal Strength of IRAEs Following Tisagenlecleucel Treatment Based on HLT Classification.

HLT_Name	N	%	ROR_025_	IC_025_
BACTERIAL INFECTIOUS DISORDERS				
CLOSTRIDIA INFECTIONS	26	10.53	2.01	0.65
STAPHYLOCOCCAL INFECTIONS	18	7.29	1.46	0.28
ENTEROCOCCAL INFECTIONS	10	4.05	1.78	0.40
ESCHERICHIA INFECTIONS	10	4.05	1.10	−0.09
PSEUDOMONAL INFECTIONS	10	4.05	0.57	−0.81
KLEBSIELLA INFECTIONS	9	3.64	1.22	0.00
STREPTOCOCCAL INFECTIONS	4	1.62	0.49	−1.05
ENTEROBACTER INFECTIONS	3	1.21	0.23	−1.83
VIRAL INFECTIOUS DISORDERS				
ADENOVIRAL INFECTIONS	15	6.07	2.25	0.72
INFLUENZA VIRAL INFECTIONS	12	4.86	4.99	1.29
RHINOVIRAL INFECTIONS	12	4.86	1.25	0.08
HERPES VIRAL INFECTIONS	11	4.45	0.43	−1.16
CORONAVIRUS INFECTIONS	7	2.83	0.93	−0.32
CYTOMEGALOVIRAL INFECTIONS	7	2.83	0.29	−1.61
POLYOMAVIRUS INFECTIONS	5	2.02	0.78	−0.56
ENTEROVIRAL INFECTIONS NEC	4	1.62	1.43	−0.19
FUNGAL INFECTIOUS DISORDERS				
CANDIDA INFECTIONS	11	4.45	0.58	−0.79
FUNGAL INFECTIONS NEC	10	4.05	0.28	−1.65
ASPERGILLUS INFECTIONS	4	1.62	0.11	−2.73

HLT, High-Level Term. Percentages (%) were calculated as the count of each HLT category divided by the total number of eligible HLGT records under the SOC “Infections and Infestations” (247). ROR_025_, lower limit of the 95% confidence interval for ROR; IC_025_, lower limit of the 95% credibility interval for IC. A statistically significant pharmacovigilance signal was defined as ROR_025_ > 1, IC_025_ > 0, and N ≥ 3.

## Data Availability

The datasets used and/or analyzed during the present study are available from the corresponding author (E-mail: 05861@tongji.edu.cn) on reasonable request. The data are not publicly available due to privacy constraints and restrictions on secondary data processing imposed by the FAERS database.

## Abbreviations

The following abbreviations are used in this manuscript:

tis-cel	tisagenlecleucel
r/r B-ALL	relapsed/refractory B-cell acute lymphoblastic leukemia
IRAEs	Infection-related adverse events
FAERS	FDA Adverse Event Reporting System
ROR	reporting odds ratio
IC	information component
CAR-T	Chimeric antigen receptor T-cell
OS	overall survival
EFS	event-free survival
CRS	cytokine release syndrome
ICANS	immune effector cell-associated neurotoxicity syndrome
HLH	hemophagocytic lymphohistiocytosis
AE	adverse event
SOC	System Organ Class
HLGT	High-Level Group Terms
HLT	High-Level Terms
PT	Preferred Terms
ROR_025_	the lower limit of the 95% confidence interval for ROR
IC_025_	the lower limit of the 95% credibility interval for IC
TTO	time-to-onset
